# Parameter prediction of coiled tubing drilling based on GAN–LSTM

**DOI:** 10.1038/s41598-023-37960-x

**Published:** 2023-07-05

**Authors:** Wanxing Zhang, Kai Bai, Ce Zhan, Binrui Tu

**Affiliations:** 1 Cooperative Innovation Center of Unconventional Oil and Gas, Yangtze University (Ministry of Education and Hubei Province), Wuhan, 430100 Hubei China; 2 Xi’an Key Laboratory of Tight Oil (Shale Oil) Development (Xi’an Shiyou University), Xi’an, 710065 Shaanxi China; 3 School of Computer Science, Yangtze University, Jingzhou, 430023 China

**Keywords:** Energy science and technology, Crude oil

## Abstract

With the increasing development of coiled tubing drilling technology, the advantages of coiled tubing drilling technology are becoming more and more obvious. In the operation process of coiled tubing, Due to various different drilling parameters, manufacturing defects, and improper human handling, the coiled tubing can curl up and cause stuck drilling or shortened service life problems. Circulation pressure, wellhead pressure, and total weight have an important influence on the working period of coiled tubing. For production safety, this paper predicts circulation pressure, ROP, wellhead pressure, and finger weight using GAN–LSTM after studying drilling engineering theory and analyzing a large amount of downhole data. Experimental results show that GAN–LSTM can predict the parameters of circulation pressure, wellhead pressure ROP and total weight to a certain extent. After much training, the accuracy is about 90%, which is about 17% higher than that of the GAN and LSTM. It has a certain guiding significance for coiled tubing operation, increasing operational safety and drilling efficiency, thus reducing production costs.

## Introduction

With the rapid development of modern drilling technology, the advantages of coiled tubing drilling technology are becoming more and more obvious. Coiled tubing has the characteristics of high strength and toughness in physical structure, and it also has the advantages of high mobility, safety and environmental protection. Therefore, it is widely used in oil and gas field service industry such as drilling, completion and logging. As coiled tubing is relatively a kind of hose, problems such as curling and jamming may occur during operation, triggering the generation of physical defects of coiled tubing, thus reducing the service life of coiled tubing. In this paper, we predict the drilling parameters of continuous tubing by deep learning algorithm to increase the service life of coiled tubing, reduce the production cost and improve the oil productivity. There is a paucity of research combining machine learning techniques with coiled tubing drilling techniques. Therefore, the integration of deep learning algorithms and coiled tubing drilling technology is a highly exploratory and valuable process. In this process, deep learning algorithms for traditional drilling parameter prediction need to be applied to coiled tubing drilling parameter prediction methods.

Currently, deep learning algorithms are widely used in conventional drilling. For example, ANN, BP neural network model, CNN model and ACO have achieved excellent results in prediction and optimization of drilling parameters (Full abbreviations are detailed in Table 1). After reviewing relevant information. Shao-Hu Liu et al. developed a new theoretical model for the problem that coiled tubing is prone to low circumference fatigue failure during operation. With this theoretical model, it was found that the reel radius, OD, and internal pressure are important parameters affecting the fatigue life of coiled tubing^1^. Wanyi Jiang et al. determined the optimal ROP by combining an artificial neural network (ANN) and an ant colony algorithm (ACO). The validity of the optimal ROP is then tested by comparing the Bayesian regularized neural network with the ROP-modified Warren model^2^. Chengxi Li and Chris Cheng applied Savitzky-Golay (SG) smoothing filter to reduce the noise in the original data set. The IGA is then used to maximize the ROP by matching the optimal ANN input parameters and the best network structure^3^ (Full abbreviations are detailed in Table 1). Cao Jie et al. analyzed the feature values affecting ROP based on feature correlation and relative importance by applying a feature engineering approach. Thus, the manual input feature parameters based on physical correlation are reduced from 12 to 8, which substantially simplifies the network model^4^. Huang et al. improved the robustness of the model by integrating the particle swarm optimization algorithm and LSTM so that the model can adapt to the complex variation pattern of oil and gas production capacity (Full abbreviations are detailed in Table 1). And it was found that the performance of LSTM is much higher than that of ordinary neural networks in time series data^5^. Liu et al. proposed a learning model that integrates LSTM and an integrated empirical model and used a genetic algorithm to determine the hyperparameters of LSTM, which can greatly improve the accuracy of the model prediction. The results show that the method exhibits very good generalization performance in terms of accuracy in predicting well production^6^.

**Table 1:** Abbreviation table.

Abbreviation	Full name
LSTM	Long Short-Term Memory
GAN	Generative Adversarial Network
ANN	Artificial Neural Network
RNN	Recurrent Neural Network
Bp	Back Propagation
ACO	Ant Colony Optimization
IGA	Improved Genetic Algorithm
OD	Outside diameter
ROP	Rate of penetration

The current difficulty in using neural networks for coiled tubing research is in two areas. One is the data, the drilling data has a confidentiality agreement can't be easily used to study, and the amount of data is huge, complex and inaccurate. The second is the selection of neural network, because the downhole data is a set of sequence data, the connection between the data before and after is relatively large, only through the RNN model to achieve better prediction effect. Therefore, after referring to Koochali's study of using GAN to predict sequential data^7^ (Full abbreviations are detailed in Table 1). In this paper, we propose a GAN and LSTM fusion model for prediction of circulation pressure, wellhead pressure, ROP and total weight data, which solves the problems arising from RNN when predicting multiple parameters and data size is too large. The experimental results show that the accuracy of the prediction of the GAN–LSTM model is around 90%.

## GAN and LSTM fusion

### Generative adversarial network

**Figure 1:**
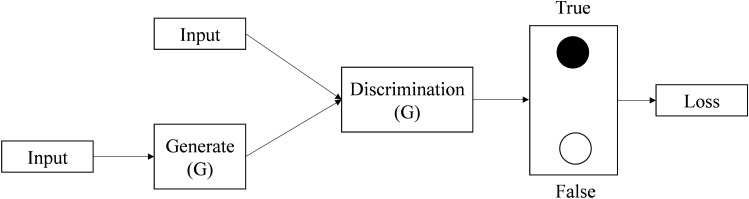
GAN model structure.

The generative adversarial network consists of a generator model (G) and a discriminator (D). The role of the generative model is to capture the data distribution and generate new data. The role of the discriminant model is to determine whether the data is real data or data generated by the generative model. Its basic structure is shown in Fig. 1.

The training set data vector Z-p(z) is used as the input to the generative model, The new data G(z) is generated after the generator network G. The input of the discriminant model D is either a real data sample or the sample G(Z) generated by the generator network. The discriminator network model is trained to determine whether its input is from the sample of real data or from the sample generated by the generator model. Then, the generator model is trained by the already trained discriminator model to generate data that more closely matches the real data distribution to deceive the discriminator. The two models play each other and are trained alternately to reach an optimal equilibrium point. At this point the generative model is able to generate data that is closest to the real data, The discriminator model cannot distinguish whether the data comes from real data or generated data. The GAN model is trained with a loss function of the following form:

1 $$\xi_{GAN} (G,D) = {\rm E}_{x \sim pdata} [\log D(x)] + {\rm E}_{z \sim p(z)} [\log (1 - D(G(z)))]$$

### Long short-term memory

**Figure 2:**
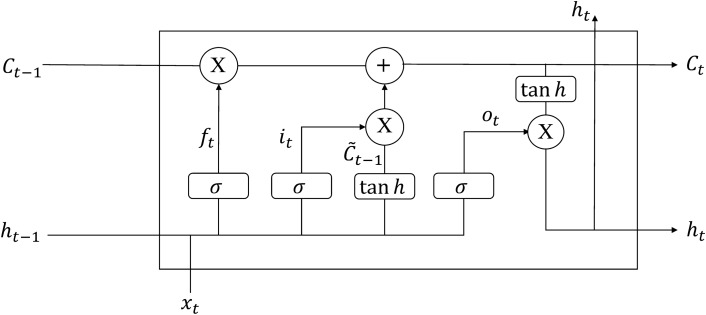
LSTM neuron internal structure.

Long Short-Term Memory (LSTM), which belongs to Recurrent Neural Network, LSTM is a modified version of a recurrent neural network. The original RNN has a hidden layer with only one state H. RNNs are very sensitive to short-term inputs and relatively weak for long-term inputs. At this point, a state C is added to the RNN, so that the RNN keeps a long-term state, thus constituting a long-short time memory network. It is usually used to process data sets with time series. LSTM can better capture the long-term dependencies between data. LSTM remembers the values in an arbitrary time interval by introducing a memory unit. Simultaneous use of input gates, output gates, and forget gates to regulate the flow of information in and out of the memory cell. Effectively solves the problem of gradient disappearance or gradient explosion of recurrent neural networks when the data size is too large. The structure of the neural unit of the LSTM is shown in Fig. 2:

First is the forgetting threshold layer. This layer is used to determine what data is to be forgotten. The forgetting gate produces a value 0~1 through the output of the previous neuron and an input variable after a sigmoid operation. The part of information near 0 will be forgotten, Instead, it continues to pass on in the united state again. This determines how much information is missing from the previous state $C_{t - 1}$.

2 $$f_{t} = \sigma (W_{f} \cdot [h_{t - 1} ,x_{t} + b_{f} ])$$

The function of the input threshold is to update the status of the old unit, this layer executes the information added or forgotten by the previous layer. A new candidate memory unit is obtained through the tanh layer. Update the previous state to $C_{t}$ under the action of the input layer.

3 $$i_{t} = \sigma (W_{i} \cdot [h_{t - 1} ,x_{t} + b_{i} ])$$

4 $$\tilde{C}_{t} = \tanh (W_{c} \cdot [h_{t - 1} ,x_{t} + b_{c} ])$$

5 $$C_{t} = f_{t} \cdot C_{t - 1} + \tilde{C}_{t} \cdot i_{t}$$

Finally, the output threshold determines what value is output. A sigmoid layer to determine which outputs are needed, then pass a $\tanh$ layer to get a value between $- 1 \sim 1$. Multiply this value with the sigmoid value to determine the final output value.

6 $$O_{t} = \sigma (W_{o} [h_{t - 1} ,x_{t} + b_{o} ])$$

7 $$h_{t} = O_{t} * \tanh (C_{t} )$$

### GAN–LSTM converged network

When a coiled tubing jam occurs at the bottom of the well, it causes changes in parameters such as bottom-hole pressure, ROP, circulation pressure, and total weight. And then cause a change in the wellhead pressure and flow rate. Data for these parameters can be measured by sensors on the surface and downhole. These datasets are used to build deep-learning models to predict drilling parameters such as total weight and ROP^8–10^.

**Figure 3:**
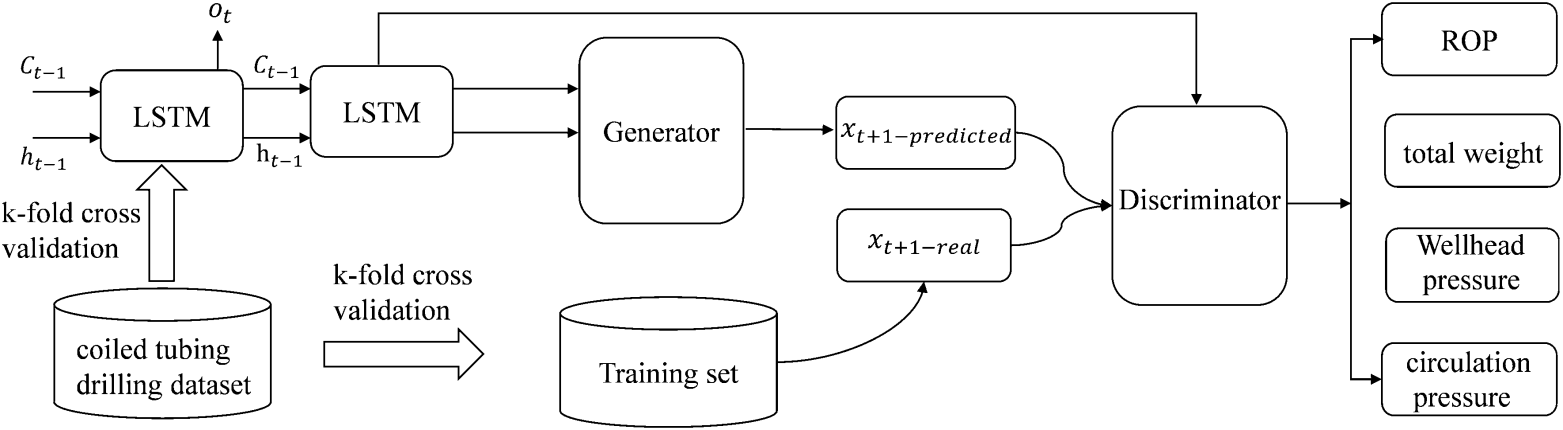
Structure of GAN–LSTM model.

Drilling history data is a typical time series data, with the characteristics of large data volume and large correlation between before and after data. Therefore, a recurrent neural network model can be used to predict drilling parameters. The disadvantage of recurrent neural networks is that they are prone to the problems of gradient disappearance and gradient explosion, resulting in poor generalization of the model^11–13^. The properties of LSTM can compensate for the problems of recurrent neural networks in terms of a gradient. When the output of the LSTM is multiple variables, the accuracy of the model prediction is significantly lower than that of the model whose output is a single variable. That is, as the dimensionality of the output data increases, the prediction accuracy decreases. And the error rate of the model increases as the depth of the predicted parameters increases^14–16^. In order to solve the above two problems, the generative model of GAN can be used to optimize the LSTM. With the powerful generative model in GAN, the low-dimensional data output from LSTM is used as the input to the generative model of GAN. The ultimate goal is to predict multiple variables and also to avoid the problem that the model accuracy decreases when the dimensionality of the output data increases. The GAN network model consists of a generative network model and a discriminative network model. The GAN–LSTM fusion network needs to use the generative network model of GAN, so the GAN and LSTM need to be trained separately during the training. The model structure of GAN–LSTM is illustrated in Fig. 3.

- *Step 1* Divide the original feature variables.
  A part of the variables is predicted by LSTM and the others are predicted by GAN. The LSTM part of the model was analyzed and experimentally attempted to predict total weight and ROP, and the GAN part predicted wellhead pressure and circulation pressure.
- *Step 2* Train the two models separately.
  LSTM: Input: Well depth, circulation pressure, wellhead pressure, ROP, and total weight. Output: ROP and total weight.
  GAN: Inputs: Well depth, circulation pressure, wellhead pressure, ROP, total weight. Output: Wellhead pressure and circulation pressure.

## Parameter prediction for coiled tubing drilling

### Data source

Based on the historical data of a single directional well in the west Sichuan area, the data from selected well sections were screened in six times, and these data were partitioned into a training set and a test set for model training by cross-validation. As shown in Table 2:

**Table 2:** Data classification.

Serial number	Original dataset	Training set	Test set
1	1,005,195	556,064	139,016
2	1,008,134	565,200	141,300
3	1,001,753	571,374	126,972
4	1,000,586	510,298	160,082
5	9,986,504	589,370	149,500
6	1,003,540	585,400	136,897

### Data processing

**Figure 4:**
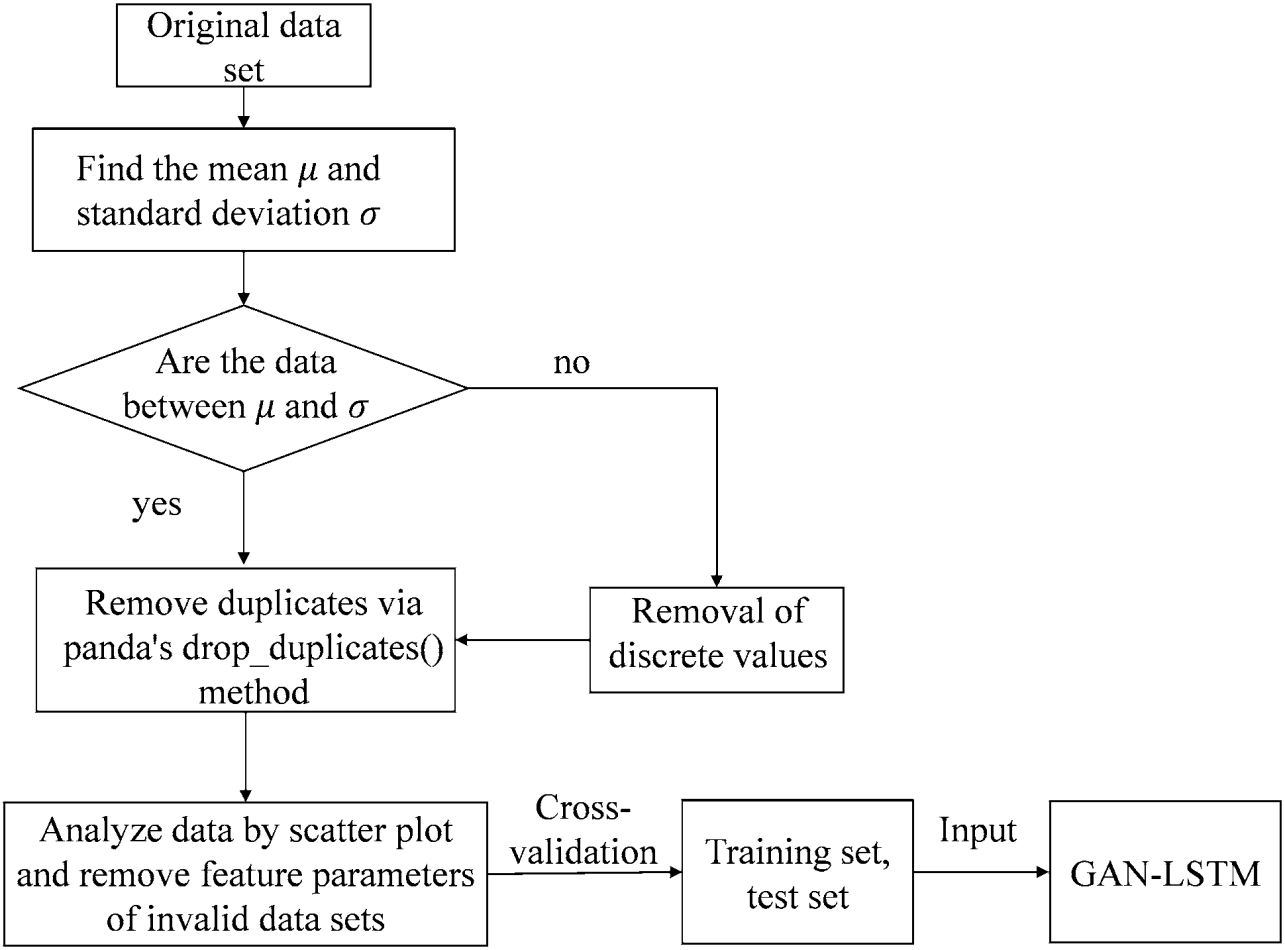
Detailed data flow chart.

Most of the downhole data is measured by sensors and therefore generates a lot of discrete, duplicate and missing data. These data can cause the overall error of the model to become larger and affect the generalization ability of the model, so some cleaning operations need to be performed on the data^17–19^. For example, removing discrete and duplicate data by clustering method, filling missing values by mean interpolation method, etc. As Fig. 4 shows the flowchart of data cleaning process.

The comparison of data samples before and after data cleaning is shown in Table 3.

**Table 3:** Comparison of data samples before and after data cleaning.

	Total weight	ROP	Wellhead pressure	Circulation pressure
Missing value or 0 value	111,293	158,004	97,840	164,095
Repeat value	196,822	142,596	197,851	146,895
Before cleaning	1,003,195	1,007,100	1,001,055	1,008,590
After cleaning	695,080	706,500	705,400	697,600

**Figure 5:**
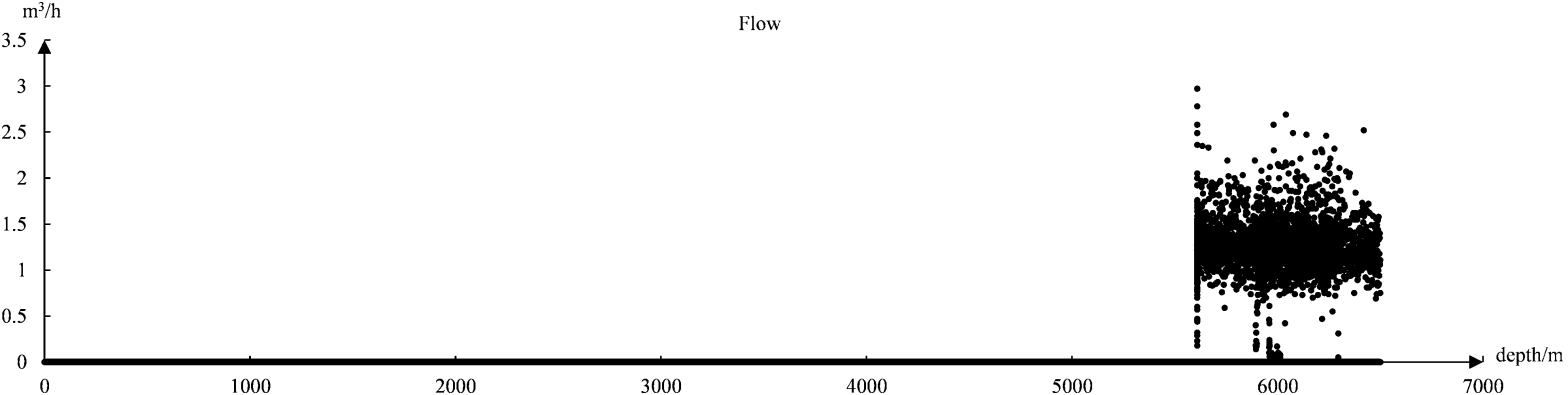
Flow data distribution chart.

**Figure 6:**
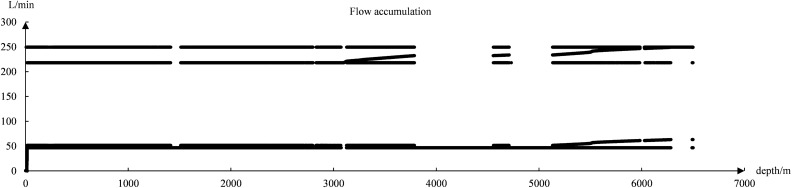
Cumulative flow data distribution.

The dataset after cleaning has 11 data features, among which 4 feature values have 90% overlap in the data. For example, the data of ROP and ROP_1 are almost the same, and after removing these 4 feature values, only 7 valid feature values are retained. The accuracy of the model is only about 50% when the data set with 7 feature values is trained. In the analysis of the data of flow and flow accumulation, it was found that. The data of the flow had 70% of 0 values and 30% of stacked duplicate values, as shown in Fig. 5. Most of the data for flow accumulation had duplicate values, as shown in Fig. 6. Therefore, after excluding the features of flow and flow accumulation. The accuracy of the model is improved by about 30%. The analysis of Eqs. 8, 9, 10, 11 and the data shows a strong correlation between the remaining five data features, which are Well depth, circulation pressure, wellhead pressure, ROP, and total weight.

8 $$v_{pc} = \frac{131.27}{{(5.5076)^{d} \times 60^{\lambda } \times (10.26)}} \times w_{s}^{d} \times n_{r}^{\lambda } \times HP_{e}^{f} \times e\vartriangle \rho_{d} (\rho_{d} - 1.15)$$

9 $$HP_{e} = 3P_{bs} \times \frac{{d_{1}^{4} + d_{2}^{4} + d_{3}^{4} }}{{(d_{1}^{2} + d_{2}^{2} + d_{3}^{2} )^{2} }}$$

In the formula: $v_{pc}$: ROP, m/h; d: drilling pressure index (d = 0.5366 + 0.1993kd), unfactored quantity; kd: Rock drillability grade value; λ: rotational speed index (λ = 0.9250–0.0375kd), uncaused quantity; f: formation hydraulic index (f = 0.7011–0.05682kd), uncaused quantity; W_s_: drilling pressure per unit bit diameter (specific drilling pressure), KN/mm; n_r_: rotational speed, r/min. HP_e_: nozzle equivalent specific water power, W/mm^2^. $\Delta \rho_{d}$: drilling fluid density difference coefficient (0.97673kd–7.2703), unfactored quantity; $\rho_{d}$: density of drilling fluid, g/cm^3^. $P_{bs}$: specific water power of the drill bit, W/mm^2^. d_1,_ d_2_, d_3_: are the drill nozzle diameters, mm, respectively.

10 $$\Delta P_{pa} = k_{pa} \times L_{p} \times Q^{1.8}$$

In the formula: $\Delta P_{pa}$: drill pipe external circulation pressure loss, MPa. k_pa_: coefficient of pressure loss in external circulation of drill pipe, unfactored quantity; L_P_: drill pipe length, m;

Q: flow rate, m^3^/h;

11 $$P_{s} = \Delta P_{b} + \Delta P_{g} + \Delta P_{cs}$$

In the formula: $P_{s}$: actual pumping pressure of the drilling pump, MPa; $\Delta P_{b}$: pressure drop of the drill nozzle, MPa; $\Delta P_{g}$: pressure loss of surface pipe sink, MPa; $\Delta P_{cs}$: circulation system pressure loss, MPa.

In the construction of the data set, the well-depth data are used as index values in the well-depth. 200 well-depth data were used as sample X (depth matrix)^20–22^. For example, the data from 1 -200 m were used to predict data such as total weight and ROP for 201-400 m. Predicting parameters such as total weight and ROP, extends the life of coiled tubing and increases operational safety and drilling efficiency.

The data of various parameters in the well are prone to some large differences in values due to different physical quantities. It causes great difficulties for model building and affects the generalization ability of the model to a great extent. Therefore, this paper normalizes the data by the commonly used Min–Max Feature scaling method. This method can simply and brutally deflate the data to between [0,1]. The formula for this method is as follows.

12 $$Z_{ij} = \frac{{x_{ij} - \min (x_{i} )}}{{\max (x_{i} ) - \min (x_{i} )}}$$

### Prediction results of different models

In this paper, after data cleaning and data normalization, we obtain valid data for five relevant features such as circulating pressure, wellhead pressure, ROP, depth, and total weight. The GAN–LSTM model is used to train the dataset with these five features data. "Mape" is the training set loss rate and "Val_mape "is the test set loss rate. The specific network parameters of GAN–LSTM are shown in Table 4.

**Table 4:** LSTM-GAN model parameters.

Layer	Layer type	Output dimension	Activation function	Related parameters
1	LSTM	32	Leaky_relu	Mape,Val_mape
2	Dropout	–	–	–
3	LSTM	64	relu	Mape,Va–l_mape
4	Dropout	–	–	–
5	LSTM	32	Leaky_relu	Mape,Val_mape
6	Dense	32	relu	–
7	Dense	16	relu	–
8	Dense	1	–	–

**Figure 7:**
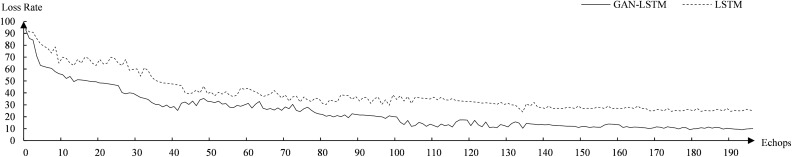
Convergence diagram of total weight loss rate.

Figure 7 shows the process of error rate change when predicting the total weight for the network model of GAN–LSTM fusion and the LSTM network model. It can be seen from Fig. 7 that the difference in error rate between the two models is small in the initial stage, and the GAN–LSTM continues to decline and stabilize after 50 rounds. The LSTM network model error rate showed large fluctuations in the early stage and gradually stabilized after 130 rounds. The error rate of the GAN–LSTM network model gradually stabilizes at around 10% after training.

**Figure 8:**
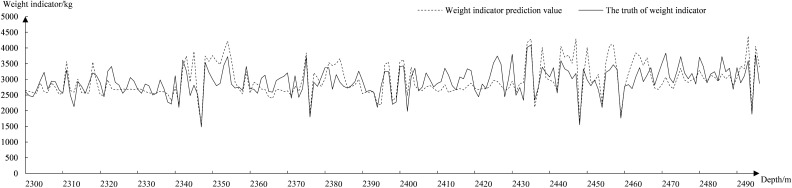
Fitted graph of GAN–LSTM predicted total weight.

**Figure 9:**
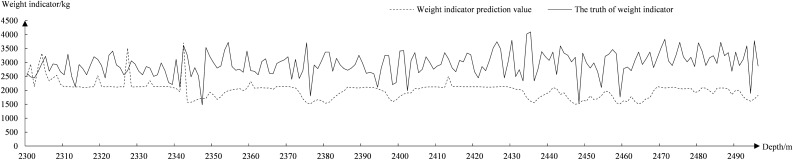
Fitted graph of LSTM predicted total weight.

Figure 8 shows the fitted curves of the GAN–LSTM model to predict the total weight. The GAN–LSTM model fits relatively stable in most cases, and the average difference between the predicted and true values is around 100. The influence of some hyperparameters causes a relatively large fluctuation between 2400 and 2470 m. Figure 9 shows the fitted curves of the LSTM model to predict the total weight. The average difference between the predicted and true values of the LSTM model in the previous section is around 200. After 2400 m the average difference of the LSTM model predictions is around 400 due to the error rate only converging to about 20%.

**Figure 10:**
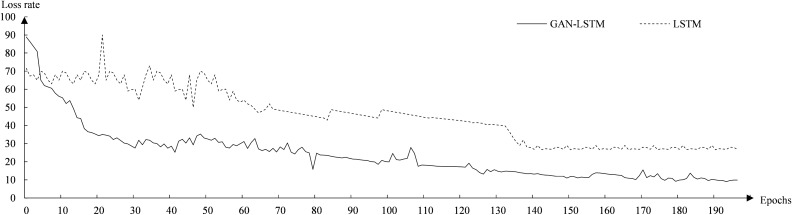
Convergence diagram of ROP loss rate.

Figure 10 shows the loss rate variation process of the GAN–LSTM network model and the LSTM network model in predicting the ROP. From Fig. 10, we can see that the error rate of the GAN–LSTM model starts to converge downward from about 90%, and the front converges relatively fast. The error rate of the GAN–LSTM model continued to decrease and stabilized at about 10% after 130 rounds. The error rate of the LSTM network model converges downward from about 70%, and there is a fluctuation of about 10% in the error rate in the first period. The error rate of the LSTM model gradually stabilized at about 27% after 140 rounds.

**Figure 11:**
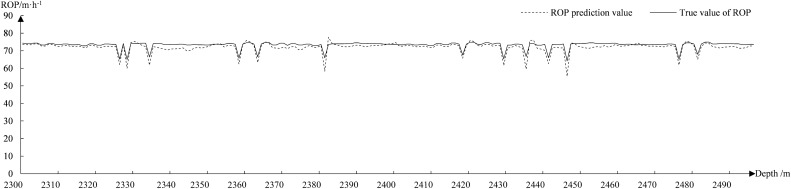
Fit of GAN–LSTM to predict ROP.

**Figure 12:**
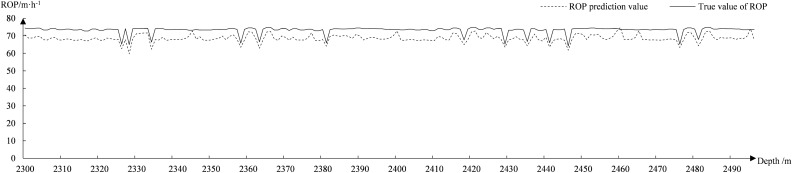
Fit of LSTM to predict ROP.

Figure 11 shows the fitted curve of the GAN–LSTM predicted ROP. The GAN–LSTM model is a relatively stable fit in most cases, and the average difference between the predicted and true values is around 1.5 m/h. The fluctuation between 2400 and 2470 m is around 3 m/h due to some hyperparameters. Figure 12 shows the fitted curves of the LSTM predicted ROP. The error rate of the LSTM model predicting ROP eventually converges only to about 27%, resulting in large fluctuations in most of the curves. The average difference between the predicted and true values is around 6 m/h.

## Conclusion

1. By combining the advantages of GAN and LSTM, a GAN–LSTM based coiled tubing drilling parameter prediction model is developed. The model consists of two parts, one part to predict wellhead pressure and circulation pressure by GAN, and one part to predict ROP and total weight by LSTM. The fusion of GAN and LSTM enhances the stability of LSTM in predicting multiple parameters, which effectively improves the generalization performance of the prediction model compared to the traditional ANN.
2. By eliminating duplicate and discrete data from the original dataset and filling in the missing data. The size of the original dataset was reduced from 1 million to 700,000 items. And based on the cleaned original dataset, four duplicate feature parameters and two feature parameters with more data 0 values and duplicate values were removed, which reduced the dimensionality of the dataset from 11 to 5 dimensions. Training with the processed dataset reveals that the accuracy of GAN–LSTM model after data cleaning is improved by 10% compared with that before cleaning.
3. A comparison of the training results of GAN, LSTM and GAN–LSTM models reveals that the average loss rate of the model with GAN–LSTM is at 10%, which is better than 25% for GAN and 28% for LSTM.

## Data Availability

Many thanks to the editors for their attention and recognition of our research work. We appreciate the SCI journal's request for data sharing but due to our lab's confidentiality agreement, we are unable to provide raw data. If the editors and reviewers have questions about specific data, we will do our best to provide more detailed explanations and clarifications. If anyone would like to obtain data from this study, please contact Dr. Bai at baikai@yangtzeu.edu.cn.

## List of symbols

$$v_{pc}$$: ROP (m/h)
d: Drilling pressure index (d = 0.5366 + 0.1993kd), unfactored quantity
kd: Rock drillability grade value
λ: Rotational speed index (λ = 0.9250–0.0375kd), uncaused quantity
f: Formation hydraulic index (f = 0.7011–0.05682kd), uncaused quantity
W_s_: Drilling pressure per unit bit diameter (specific drilling pressure) (KN/mm)
n_r_: Rotational speed (r/min)
HP_e_: Nozzle equivalent specific water power (W/mm^2^)
$\Delta \rho_{d}$: Drilling fluid density difference coefficient (0.97673kd–7.2703), unfactored quantity
$\rho_{d}$: Density of drilling fluid (g/cm^3^)
$P_{bs}$: Specific water power of the drill bit (W/mm^2^)
d_1,_ d_2_, d_3_: Are the drill nozzle diameters, mm, respectively
$\Delta P_{pa}$: Drill pipe external circulation pressure loss (MPa)
k_pa_: Coefficient of pressure loss in external circulation of drill pipe, unfactored quantity
L_P_: Drill pipe length (m)
Q: Flow rate (m^3^/h)
$P_{s}$: Actual pumping pressure of the drilling pump (MPa)
$\Delta P_{b}$: Pressure drop of the drill nozzle (MPa)
$\Delta P_{g}$: Pressure loss of surface pipe sink (MPa)
$\Delta P_{cs}$: Circulation system pressure loss (MPa)
